# Evaluation of Male Fertility-Enhancing Activities of Water Seed Extract of* Hunteria umbellata* in Wistar Rats

**DOI:** 10.1155/2019/7693010

**Published:** 2019-08-14

**Authors:** Adejuwon Adewale Adeneye, Joseph Abayomi Olagunju, Babatunde Adekunle Murtala

**Affiliations:** ^1^Department of Pharmacology, Therapeutics & Toxicology, Faculty of Basic Clinical Sciences, Lagos State University College of Medicine, 1-5 Oba Akinjobi Way, G.R.A, Ikeja 100001, Lagos, Nigeria; ^2^Department of Medical Biochemistry, Faculty of Basic Medical Sciences, Lagos State University College of Medicine, 1-5 Oba Akinjobi Way, G.R.A, Ikeja 100001, Lagos, Nigeria; ^3^Department of Physiology, Faculty of Basic Medical Sciences, Lagos State University College of Medicine, 1-5 Oba Akinjobi Way, G.R.A, Ikeja 100001, Lagos, Nigeria

## Abstract

**Background:**

In this study, the male fertility-enhancing activity of 100, 200, and 400 mg/kg/day of* Hunteria umbellata* water seed extract (*HU*) in Wistar rats was studied for 60 days. In doing this, effect of repeated doses of* HU* was studied on the weight gain pattern, gonadosomatic index (GSI), serum follicle stimulating hormone (FSH), luteinizing hormone (LH), testosterone (TS), prolactin (PRL), and estradiol (ES)} as well as testicular antioxidant status of the treated rats as a way of elucidating the mechanism(s) of action of* HU*.

**Method:**

Thirty-six (36) male Wistar rats were randomly divided into six groups (I-VI) of six rats per group. Group I rats were gavaged with 10 ml/kg/day of distilled water and served as an untreated control; Group II rats were gavaged with 0.3 mg/kg/day of clomiphene in distilled water; Groups III-V rats received 100 mg/kg/day, 200 mg/kg/day, and 400 mg/kg/day of* HU*, respectively, and Group VI rats received 20 mg/kg/day of Vitamin C all in distilled water. All treatments were for 60 days after which the treated rats were humanely sacrificed. Sera of blood samples were processed for the above stated hormonal profile. Similarly, testicular tissues obtained were processed for semen analysis and complete antioxidant profile of the* HU*-treated testicles by assaying for superoxide dismutase (SOD), catalase (CAT), and glutathione (GSH), glutathione reductase (GSR), glutathione peroxidase (GSH-Px), and Thiobarbituric Reactive Species (TBARS).

**Results:**

Prolonged treatments with 100 mg/kg/day, 200 mg/kg/day, and 400 mg/kg/day of* HU* for 60 days induced dose dependent reductions in weight gain pattern with the most significant (p<0.001) effect recorded with the highest dose of* HU*. Conversely, significant (p<0.001) increase was recorded for GSI at the same* HU* dose. Clomiphene and* HU* also induced significant (p<0.01, p<0.001) dose dependent increases in the total sperm count, %live sperm, but reverse effects on %dead sperm and %abnormal sperm. On the hormonal profile, oral treatment with 100 mg/kg/day, 200 mg/kg/day, and 400 mg/kg/day of the extract induced profound (p<0.05, p<0.01, and p<0.001) dose related increases in the sera TS, LH, and FSH while it caused reverse effect on serum PRL but caused no significant alterations in the serum ES levels. Similarly, oral treatment with vitamin C and 100-400 mg/kg/day of* HU* induced profound (p<0.05, p<0.01, and p<0.001) increases in the antioxidant enzyme activities.

**Conclusion:**

Overall, prolonged oral treatment with 100-400 mg/kg body weight of* HU* for 60 days significantly improved sperm function which was mediated via enhanced spermatogenesis, steroidogenesis, and antioxidant mechanisms.

## 1. Background

Infertility is a major public health issue affecting one out of five every married couple worldwide, with approximately 30% of the condition attributable to male factors [1]. It is on records that several factors can interfere with the process of spermatogenesis and reduce sperm quantity and quality with some of the identifiable causal factors being ischemic heart disease, diabetes mellitus, chronic liver diseases, cigarette smoking, agrochemical run-offs, air pollutants, and hypovitaminosis [2]. However, previous study has reported that regular intake of antioxidants and vitamins such as vitamins A, B, C, and E improves testicular blood barrier stability and protects sperm DNA damage from endogenous oxidative stress resulting from the activities of highly reactive free radicals generated within the body [3].

Male infertility can manifest in the form of premature ejaculation, hypoactive sexuality, erectile dysfunction, oligospermia, azoospermia,* etc*., but its most common manifestation form is as oligospermia. In treating this condition, testosterone and other forms of hormone replacement therapy are often clinically employed due to their ability to stimulate/enhance sexual appetite in hypogonadal male patients [4, 5]. However, despite the proven efficacy of this replacement therapy in the management of hypoactive sexual desire [6], many patients still prefer to use natural plants because of the attendant undesirable side-effects associated with these hormonal therapies. Two examples of popular male fertility-promoting herbs are* Panax *spp. (ginseng) and* Lepidium meyenii *(Maca) which are reputed for their supposed aphrodisiac- and spermatogenesis-enhancing effects [7–9].

*Hunteria umbellata* (K. Schum.) Hallier f., belonging to Apocynaceae family, is a tropical rainforest tree that is commonly used in the African folkloric medicine to treat human diseases such as blood deficiencies, infections, swellings, diabetes mellitus, and obesity [10–12]. It is known as “Demouain” (in French) and “Abeere” (in Yoruba dialect) (Southwest Nigeria) [11–13]. In Southwest region of Nigeria, water infusion of* Hunteria umbellata* dried seed is reputably used in the local management of diabetes mellitus and obesity [12, 14].* Hunteria umbellata* water seed extract (*HU*) of the plant has been reportedly used to effectively control blood glucose and glycosylated hemoglobin concentration in types 1 and 2 models of drug-induced hyperglycemic Wistar rats [12, 14] with its alkaloid content implicated for the observed biological effect [15]. The same plant seed extract was also reported to possess antiobesity and antihyperlipidemic effects in experimental models of hyperlipidaemia which was mediated via* de novo* inhibition of cholesterol and triglyceride biosynthesis [16]. In addition, the acute, chronic, and reverse oral toxicity studies of* HU* have shown it to be relatively safe [12, 17].

Recent ethnobotanical survey conducted among selected Ijebu herbal practitioners in Ogun State (Southwest Nigeria) revealed that* HU* also has a wide application in the indigenous management of infertility (unpublished data). Despite this folkloric use, there are no scientific reports to either validate or refute this folkloric claim. More so, chronic oral toxicity and toxicity reversibility studies of* HU* have shown it to induce profound proliferation of spermatogenic primordial, Sertoli and Leydig's cells in the testicular tissues of extract-treated rats [17]. Similarly,* HU* has been reported to possess antioxidant and free radical scavenging properties mediated primarily by its alkaloid content [18]. Thus, this study was designed at evaluating the effects of chronic oral treatment with 100, 200, and 400 mg/kg of* HU* on body weight, gonadosomatic index, and semen parameters of male Wistar rats treated for 60 days. In addition, effects of HU oral treatment on the complete antioxidant enzymes system [superoxide dismutase (SOD), catalase (CAT), and glutathione (GSH), glutathione reductase (GSR), glutathione peroxidase (GSH-Px), and Thiobarbituric Reactive Species (TBARS)] of treated rat testicles as well as its effect on serum testosterone (TS), luteinizing hormone (LH), follicle stimulating hormone (FSH), prolactin (PRL), and estradiol (ES) were evaluated.

## 2. Methods

### 2.1. Plant Material Collection and Identification

After plant identification and deposit of voucher specimen were done as earlier described by Adeneye and Adeyemi [12], eight (8) ripe* Hunteria umbellata* fruits were freshly harvested from the deciduous forest of Odorasanyin District of Ijebu-Igbo in Ogun State, Nigeria, in the months of July-August, 2016. Collected fruits were cut into pieces and their fresh seeds were rinsed generously under a running tap water and air-dried at room temperature (25 ± 1°C) for 1 month, protected from direct heat and sunlight. The dried seeds were milled using Laboratory Hammer Mill in the Pharmacognosy Department, Faculty of Pharmacy, University of Lagos, Idi-Araba, Lagos. The milled sample was preserved in a water- and air-proof container and stored at 4°C.

### 2.2. Aqueous Extraction Process

50 g of milled seeds of* Hunteria umbellata* was soaked in 500 ml of distilled water and kept in the refrigerator for 72 hours. The solution was repeatedly stirred using magnetic stirrer for 6 hours before it was filtered with sterilized white handkerchief-packed filter funnel. The filtrate obtained was then completely air-dried in aerated oven preset at 40°C resulting in a deep brown, sweet-smelling solid residue (*HU*). This procedure was repeated 9 more times and the residues were pooled into a water- and air-proof container and stored in the freezer at -4°C to prevent HU from decomposing.

### 2.3. Experimental Animals

After an Institutional Ethical Approval on the Use of Experimental Animals was obtained, thirty six 12-14-week-old male Wistar rats weighing between 220 g and 250 g were obtained from Bayo Farms, Sango-Otta, Ogun State, Nigeria, and housed in the Animal House of Lagos State University College of Medicine for acclimatization for 14 days. While being acclimatized, rats were fed with standard rat chow and tap water* ad libitum* and maintained at standard laboratory conditions (12/12 hour light-dark periodicity, temperature: 23-26°C and 40-50% relative humidity) as prescribed by the United States National Institute for Health [19]. Two days to the commencement of the animal experiment, rats were randomly allotted into 6 groups of 6 rats per treatment group such that the weight differences within and between treatment groups do not exceed ±20% of the average weight of the rat sample population, respectively.

### 2.4. Treatment of Rats

Thirty six (36) male Wistar rats of proven fertility were divided randomly into 6 groups of 6 animals each and their oral treatments per group were as follows:

  Group I: 10 ml/kg of distilled water/day for 60 days.
  Group II: 0.3 mg/kg/day clomiphene in distilled water for 60 days.
  Group III: 100 mg/kg/day* HU* in distilled water for 60 days.
  Group IV: 200 mg/kg/day* HU* in distilled water for 60 days.
  Group V: 400 mg/kg/day* HU* in distilled water for 60 days.
  Group VI: 20 mg/kg/day of vitamin C in distilled water for 60 days.

### 2.5. Body Weight Measurement

Rat body weights were measured using digital Mettler weighing balance and values obtained recorded a day prior to commencement of the experiment, biweekly during the treatment period, and on the last day of the experiment.

### 2.6. Calculation of Gonadosomatic Index (GSI)

The testes and their cauda epididymis were identified, removed, and weighed and immediately fixed in Bouin's solution for morphometric study. The gonadosomatic indices were calculated as follows:

 
{testes weight ÷ body weight} x 100 [20]

### 2.7. Hormonal Assays

The testosterone level in the serum was estimated by radioimmunoassay (RIA). The assays were performed using commercially available kits (Diagnostic Products Company, Los Angeles, CA, USA). All samples were run in the same assay period. The within assay variation was 5·5% while the sensitivities of the testosterone assay was 8 ng/ml. Sera FSH, LH, PRL, and ES levels were measured by immunoradiometric assay (IRMA) in solid phase also using commercially available kits (Diagnostic Products Company, Los Angeles, CA, USA).

### 2.8. Preparation of Semen Sample for Analysis

On day 61, the rats were euthanized with inhaled diethyl ether and a longitudinal surgical incision along the scrotal raphe and scrotal septum was made to expose the testes and its epididymis. The epididymis was freed from the adhering fat and connective tissues. The left epididymis was collected, weighed, and cut at the distal end using a clean surgical blade. Cauda epididymis (100 mg) was gently minced with glass rod without damaging the tissue into 5 ml of 0.9% NaCl [21].

### 2.9. Semen Analysis for Motility, Count, and Morphology

Complete semen analysis to determine progressive motility, count, and morphology was done in clean Neubauer's haemocytometer counting chamber under its cover-slip using the method of Amman [21].

Number of sperms per cauda epididymis was calculated as follows:

 
{Mean count x 50} ÷ {0.01 x 0.01}

### 2.10. Estimation of Testicular Reduced Glutathione Levels

In determining the testicular GSH level, testicular tissue homogenate in 0.1M phosphate buffer at pH of 7.4 was processed through the procedure described by Shaik and Mehvar [22].

### 2.11. Estimation of Testicular Tissue SOD and CAT Activities

Testicular tissue SOD and CAT activities were estimated using the method of Zhang* et al.* [23] and Iwase* et al*. [24], respectively.

### 2.12. Data Analysis

Data obtained were presented as mean ± S.E.M. of six observations. Data were analyzed statistically using One-way analysis of variance on SYSTAT 10.6.* Post hoc* test was done using Student's t-test and levels of significance were considered at p<0.05, p<0.01, and p<0.001.

## 3. Results

### 3.1. Water Extraction

Water extraction of milled* Hunteria umbellata* dried seeds produced a deep brown, sweet-smelling solid residue weighing an average of 7.33 ± 0.32 g with a %yield of 14.66 ± 0.65%.

### 3.2. Effects of Oral Treatments with Clomiphene, Vitamin C, and* Hunteria umbellata* Water Seed Extract on Rat Body Weights

Repeated oral treatments with 100-400 mg/kg/day of* HU* induced significant (p<0.05, p<0.01, and p<0.001) dose related decreases in the weight gain pattern of treated rats effective from the 30^th^ day to the 60^th^ day of oral treatment when compared to the weight gain pattern of Vitamin C- and clomiphene-treated rats over the same treatment period. The most significant (p<0.001) weight reduction was observed at 400 mg/kg/day* HU* on the 60^th^ day (Table 1). However, daily oral treatment with 0.3 mg/kg/day of clomiphene for 60 days caused steady and consistent body weight increases of treated rats with the most significant (p<0.001) increase recorded on day 60 (Table 1). Similar effect was reported for 20 mg/kg/day of Vitamin C (Table 1).

### 3.3. Effect of Oral Treatment with 100-400 mg/kg/day of* Hunteria umbellata* Water Seed Extract on Rat Testicular Weight (TW) and Gonadosomatic Indices (GSI)

Repeated oral treatment with 100, 200, and 400 mg/kg* HU* induced dose related increases in the testicular weight and GSI of treated rats with significant increases (p<0.001) recorded for the 400 mg/kg/day* HU*-treated rats (Table 2). The same effects were also recorded in clomiphene- and vitamin C-treated rats (Table 2).

### 3.4. Effect of Oral Treatment with 100-400 mg/kg/day of* Hunteria umbellata* Water Seed Extract on Rat Semen Parameters

Repeated daily oral treatment with 100 mg/kg/day, 200 mg/kg/day, and 400 mg/kg/day* HU* for 60 days on semen analysis showed significant (p<0.05, p<0.01, and p<0.001) dose-related increases in the total sperm count of treated rats with the most significant (p<0.001) boost recorded in rats treated with the highest dose (400 mg/kg/day) of the extract which was comparable with what was recorded for the standard drug (0.3 mg/kg/day of clomiphene) (Table 3). Similar pattern was also recorded for % motile sperm counts (Table 3). However,* HU* had a reverse effect on the % dead sperm and % abnormal sperm counts (Table 3) while 0.3 mg/kg/day of clomiphene caused a significant (p<0.001) increase in the %abnormal sperm count when compared to untreated control (Group I) rats (Table 3).

### 3.5. Effect of Oral Treatment with 0.3 mg/kg/day of Clomiphene and 100-400 mg/kg/day of* Hunteria umbellata* Water Seed Extract on Rat's Sera TS, LH, FSH, PRL, and ES

Repeated oral treatment with 0.3 mg/kg/day of clomiphene resulted in significant (p<0.001) increases in the sera TS, LH, and FSH levels when compared to the untreated control (Group I) values (Table 4). However, 0.3 mg/kg/day clomiphene significantly (p<0.05) reduced circulating serum ES level when compared to Group I values (Table 4). Similarly, repeated treatments with graded oral doses of* HU* for 60 days resulted in significant (p<0.05, p<0.01, and p<0.001) dose related increases in the circulating serum TS, LH, and FSH levels while resulting in significant (p<0.05) reductions in serum PRL when compared to untreated control (Group I) values (Table 4). However, there were no significant alterations in the serum ES levels between the treatment groups and the untreated control group (Table 4).

### 3.6. Effect of Oral Treatment with 0.3 mg/kg/day of Clomiphene and 100-400 mg/kg/day of* Hunteria umbellata* Water Seed Extract on Rat Testicular Tissue SOD, CAT, and TBARS

Repeated oral treatments with 0.3 mg/kg/day of clomiphene did not significantly (p>0.05) alter the testicular tissue activities of SOD, CAT, and TBARS compared to those of untreated control (Group I) values (Table 5). However, repeated daily oral treatment with 100-400 mg/kg/day of* HU* caused significant (p<0.05 and p<0.001) dose related increases in the testicular tissue levels of SOD, CAT, and TBARS when compared to both untreated control (Group I) and clomiphene-treated (Group II) values (Table 5). Similarly, oral treatments with 20 mg/kg/day of Vitamin C had similar effects on the testicular tissue SOD, CAT, and TBARS recorded for rats treated with 400 mg/kg/day of* HU* (Table 5).

### 3.7. Effect of Oral Treatment with 100-400 mg/kg/day of* Hunteria umbellata* Water Seed Extract on Rat Testicular Tissue GSH, GSH-Px, and GSR

Treatments with 100-400 mg/kg/day of* HU* caused significant (p<0.05, p<0.01, and p<0.001) dose related testicular tissue levels of GSH, GSH-Px, and GSR compared to untreated control (Group I) and clomiphene-treated (Group II) values (Table 6). However, the values obtained for 400 mg/kg/day of* HU* were comparable with those recorded for Vitamin C-treated rats (Table 6). It is also worthy to note that treatments with 0.3 mg/kg/day of clomiphene had no profound alterations (p>0.05) in the testicular tissue levels of GSH, GSH-Px, and GSR (Table 6).

## 4. Discussion

Infertility is the inability of a couple of opposite sex to achieve a clinical pregnancy after 52 weeks or more of regular unprotected sexual intercourse. In other words, it is a complete failure of a sexually competent and active, noncontracepting couple to achieve pregnancy in one or more years despite regular sexual exposures [25]. Recent epidemiological data has shown that approximately 15% of married/unmarried couples are affected by infertility for which only 40-50% cases are attributable to male infertility [26] which itself could be due to either azoospermia or erectile dysfunction [27]. Identifiable factors (reversible/irreversible) influencing male fertility as reported by previous studies include drugs (such as anabolic steroid, replacement testosterone, and opiates), testicular varicocele, undescended testes, urinary tract infections, testicular tumor, hormonal imbalances, premature or retrograde ejaculation, prolonged heat exposure, obesity, older age, cigarette smoking, alcohol, heavy metals, pesticides, oxidative stress, genetic factors, and different environmental and nutritional factors [28–34]. Thus, in managing male infertility, treatment plans are designed alongside the identifiable possible cause and these interventions could be either medical or surgical [27, 35].

Apart from orthodox treatment of infertility, infertile couples are known to concomitantly use traditional/complementary medicine as therapeutic alternatives for their infertility since 25% of modern drugs are either plant-based or of plant origin [36]. More so, up to 80% of the world's population is either partially or wholly dependent on herbal products for the treatment of their medical conditions [37]. However, in evaluating pharmacological activities of male fertility-enhancing medicinal plants, scientific investigations are done* in vitro* (using culture cell lines) and* in vivo* (using experimental animals and humans) models. For examples, extracts obtained from* Vanda tessellata* [38],* Turnera diffusa* and* Pfaffia paniculata* [39],* Eurycoma longifolia* [40],* Terminalia catappa* [41],* Butea frondosa* [42],* Curculigo orchioides* [43],* Panax quinquefolius *(ginseng) [44], and* Lepidium meyenii* have been investigated and reported to enhance aphrodisiacs while extracts obtained from* Astragalus membranaceus, Asparagus racemosus, Withania somnifera, Andrographis paniculata*, and* Acanthopanax senticosus* were also reported to improve sperm parameters [45, 46].

In this study we evaluated the possible fertility-enhancing and fertility mechanism of action of* HU* in male Wistar rats for 60 days as an alternative/complimentary to existing fertility-boosting drugs bearing in mind their high cost and untoward side effects associated with their clinical use. In doing this, male Wistar rats were gavaged with graded doses (100, 200, and 400 mg/kg/day) of HU in male Wistar rats for 60 consecutive days using endpoints such as the somatogonadal index, semen analysis parameters, and gonadal hormone profile as well as the testicular tissue antioxidant profile.

Hormonal analysis of* HU*-treated rats clearly showed the extract to significantly improve circulating sera TS, FSH, and LH while it decreased circulating prolactin. Literature has shown that serum elevation in the testosterone, FSH, and LH improves sperm quality in relation to sperm count, volume, motility, and morphology [43]. FSH and LH are known to influence the fate of germ cells and this influence is mediated via their actions on specific transmembrane receptors, Follicle Stimulating Hormone receptor (FSH-R), and Luteinizing Hormone receptor (LH-R) that are overtly expressed in the Sertoli cells and interstitial Leydig cells, respectively [47–50]. Scientific evidences abound for the critical role of the LH-testosterone signaling pathway in priming and sustaining the process of spermatogenesis in the extratubular Leydig cells while FSH regulates spermatocytogenesis and spermatogenesis by influencing both the germinal epithelium and the Sertoli cells [51, 52]. Thus, interplay of FSH, LH, and testosterone strongly influences quantitative and qualitative spermatogenesis. In this study, repeated oral treatment with 100-400 mg/kg/day of* HU* stimulated significant spermatogenesis which was characterized by increased sperm volume, count, progressive motility, and improved sperm morphology. This improvement in sperm quality could have been due to improved steroidogenesis characterized by increased circulating TS, FSH, and LH which have been widely reported to enhance spermatogenesis [53]. Clomiphene like tamoxifen is a known anti-estrogenic analogue which blocks estrogen from interacting with the anterior pituitary gland to induce increased LH, FSH and TS syntheses [54]. Thus, the mechanism of fertility enhancement by HU appears to be similar to that of clomiphene.

Another notable finding of this study is the nonsignificant alteration in circulating prolactin levels for treated groups. Physiologically, prolactin suppresses LH and FSH secretion which invariably lowers circulating TS levels and decreases sexual drive. This assertion is in complete agreement with elevated FSH, LH, and TS levels in the* HU*-treated groups. Previous studies have reported that any plant with aphrodisiac/male fertility enhancing potential often profoundly lowers serum PRL and enhances circulating LH and FSH levels and by extension enhances circulating TS levels [55, 56] with which our results are in complete agreement.

Medicinal plants rich in certain secondary metabolites such as phenolic acids, flavonoids, flavones, alkaloids, and antioxidant vitamins (e.g., Vitamins A, C, D, and E) have been reported to stimulate and enhance spermatogenesis [57–61]. However, our previous studies have reported* HU* to be abundantly rich in alkaloids, flavonoids, tannins, and glycosides [12]. Thus, the relative abundance of these phytochemicals could have been responsible for the increased spermatogenesis observed in this study.

The cause of infertility in about 50% of all infertile couples is attributable to male factors either solely or partly for which oxidative stress is one of the factors [62]. Excessive production of free radicals or reactive oxygen species (ROS) has implicated sperm damage and as such ROS have been extensively studied as in the etiology of male infertility. Superoxide anion, hydroxyl radical, and hydrogen peroxide are some of the major ROS present in seminal plasma and these have been implicated in male infertility [63–66]. Under normal physiological conditions, small and negligible amount of ROS is generated by spermatozoa and this is essential for spermatozoa capacitation and acrosomal reaction. However, when ROS generation is overwhelming, it could irreversibly bind to the spermatozoa plasma membrane- and cytoplasmic-rich polyunsaturated fatty acids resulting in lipid peroxidation and cell death of spermatozoa [67].

Antioxidants including superoxide dismutase (SOD), catalase, and glutathione peroxidase (GPX) often free scavenge ROS to protect spermatozoa from the deleterious effect of the generated ROS [68, 69]. Semen is also contains a variety of endogenous nonenzymatic antioxidant molecules such as vitamins A, C, D, and E, pyruvate, glutathione, and carnitine [70]. These antioxidants compensate for the loss of sperm cytoplasmic enzymes during spermatogenesis, which in turn diminishes endogenous repair mechanisms and enzymatic defenses [71–73]. In view of the established direct relationship between male fertility and testicular antioxidant status,* HU* significantly improved the antioxidant status of the treated rat testicles as indicated by increased levels of SOD, CAT, GSH, GSH-Px, GSR, and TBARS, which are in consonance with our previous* in vitro* [18] and* in vivo* [74] findings and that recently are reported by Oboh* et al*. [10]. Thus, these results clearly demonstrate the potential of* HU* in significantly improving the spermatogenic parameters via its antioxidant mechanism. Also, literature has shown antioxidants to improve various oxidative processes including spermatogenesis and steroidogenesis [68, 75], thus lending credence to the observed spermatogenic and steroidogenic effects of* HU* in the treated rats to be attributed to its high antioxidant profile.

## 5. Conclusion

Overall, prolonged oral treatments with 100-400 mg/kg/day of* HU* for 60 days significantly improved sperm function (as measured by sperm motility, count, viability, and morphology), mediated via increased spermatogenesis, steroidogenesis, and antioxidant mechanisms.

## Figures and Tables

**Table 1:** Effect of repeated daily oral treatments with 100-400 mg/kg/day of *Hunteria umbellata* aqueous seed extract on the weight gain pattern of treated rats.

Groups	Average body weight (g) on the following:				
	Day 1	Day 15	Day 30	Day 45	Day 60
I	227.80±12.95	229.80±11.58	240.70±9.11	260.70±8.54	290.50±11.90
II	229.70±13.37	239.20±16.90	256.20±13.72	276.7±12.47^a+^	315.30±13.76^c+^
III	226.80±12.16	231.80±10.94	241.00±9.80	256.30±5.13	280.00±9.42
IV	230.20±13.56	236.00±13.61	254.50±11.29	264.80±13.08	279.20±14.95
V	225.00±14.52	226.70±12.45	234.00±13.45^a−^	242.80±12.95^b−^	243.70±11.74^c−^
VI	225.00±15.67	240.50±13.32	259.50±9.94^a+^	282.20±6.71^b+^	307.80±5.78^c+^

^a+,b+^ and  ^c+^ represent significant increases at p<0.05, p<0.01, and p<0.001, respectively, when compared to Group I values, while  ^a−,b−^ and  ^c−^ represent significant decreases at p<0.05, p<0.01, and p<0.001, respectively, when compared to Group II values.

I = 10 ml/kg/day of distilled water.

II = 0.3 mg/kg/day of clomiphene dissolved in distilled water.

III = 100 mg/kg/day of *Hunteria umbellata* aqueous seed extract dissolved in distilled water.

IV = 200 mg/kg/day of *Hunteria umbellata* aqueous seed extract dissolved in distilled water.

V = 400 mg/kg/day of *Hunteria umbellata* aqueous seed extract dissolved in distilled water.

VI = 20 mg/kg/day of Vitamin C dissolved in distilled water.

**Table 2:** Effect of repeated daily oral treatments with 100-400 mg/kg/day of *Hunteria umbellata* aqueous seed extract on the average testicular weight (TW) and gonadosomatic indices (GSI) of treated rats.

Groups	Average rat weight on day 60 (g)	TW (g)	SGI (x10^−2^)
I	290.50 ± 11.90	03.51 ± 0.32	12.07 ± 0.66
II	315.30 ± 13.76^c+^	04.27 ± 0.43^c+^	13.50 ± 0.81^b+^
III	280.00 ± 09.42	03.37 ± 0.20	12.01 ± 0.32
IV	279.20 ± 14.95	03.36 ± 0.27	12.04 ± 0.34
V	243.70 ± 11.74	03.94 ± 0.03	16.21 ± 0.88^c+^
VI	307.80 ± 5.78	04.04 ± 0.16^b+^	13.05 ± 0.38^a+^

^a+,b+^ and  ^c+^ represent significant increases at p<0.05, p<0.01, and p<0.001, respectively, when compared to Group I values.

I = 10 ml/kg/day of distilled water.

II = 0.3 mg/kg/day of clomiphene dissolved in distilled water.

III = 100 mg/kg/day of *Hunteria umbellata* aqueous seed extract dissolved in distilled water.

IV = 200 mg/kg/day of *Hunteria umbellata* aqueous seed extract dissolved in distilled water.

V = 400 mg/kg/day of *Hunteria umbellata* aqueous seed extract dissolved in distilled water.

VI = 20 mg/kg/day of Vitamin C dissolved in distilled water.

**Table 3:** Effect of repeated daily oral treatments with 100-400 mg/kg/day of *Hunteriaumbellata* aqueous seed extract on semen parameters of treated rats.

Groups	Total sperm/g cauda epi (x10^7^)	%motile sperm	%dead sperm	%abnormal sperms
I	29.58 ± 0.55	77.03 ± 0.56	22.97 ± 0.56	08.97 ± 0.41
II	50.85 ± 1.01	87.23 ± 1.44^c+^	12.77 ± 1.44^f^	14.03 ± 0.87^c+^
III	31.68 ± 0.55	78.55 ± 0.40	21.45 ± 0.40	19.12 ± 0.19^c+^
IV	37.93 ± 0.71^b+^	79.27 ± 0.62	20.73 ± 0.62	12.17 ± 0.62^c+^
V	47.38 ± 0.84^c+^	87.17 ± 1.32^c+^	16.83 ± 1.32^f^	07.67 ± 0.91^f^
VI	31.70 ± 1.03	78.22 ± 0.38	21.78 ± 0.38	10.86 ± 0.48^e^

^a+,b+^ and  ^c+^ represent significant increases at p<0.05, p<0.01, and p<0.001, respectively, when compared to Group I values, while  ^e^ and  ^f^ represent significant decreases at p<0.05 and p<0.001, respectively, when compared to Group I values.

I = 10 ml/kg/day of distilled water.

II = 0.3 mg/kg/day of clomiphene dissolved in distilled water.

III = 100 mg/kg/day of *Hunteria umbellata* aqueous seed extract dissolved in distilled water.

IV = 200 mg/kg/day of *Hunteria umbellata* aqueous seed extract dissolved in distilled water.

V = 400 mg/kg/day of *Hunteria umbellata* aqueous seed extract dissolved in distilled water.

VI = 20 mg/kg/day of Vitamin C dissolved in distilled water.

**Table 4:** Effect of repeated daily oral treatments with 100-400 mg/kg/day of *Hunteria umbellata* aqueous seed extract on serum testosterone (TS), luteinizing hormone LH), follicle stimulating hormone (FSH), prolactin (PRL), and estradiol (ES) of treated rats.

Groups	TS (ng/ml)	LH (ng/ml)	FSH (ng/ml)	PRL (ng/ml)	ES (ng/ml)
I	02.53 ± 0.04	0.66 ± 0.03	02.32 ± 0.04	03.14 ± 0.19	2.40 ± 0.06
II	05.87 ± 0.68^c+^	01.47 ± 0.44^c+^	03.06 ± 0.09^c+^	02.86 ± 0.11	2.13 ± 0.07
III	04.04 ± 0.16^a+^	00.83 ± 0.08^a+^	02.66 ± 0.03^a+^	02.82 ± 0.10	2.37 ± 0.04
IV	04.70 ± 0.15^b+^	01.16 ± 0.05^b+^	02.76 ± 0.04^b+^	02.42 ± 0.17	2.46 ± 0.10
V	06.39 ± 0.31^c+^	01.42 ± 0.05^c+^	03.25 ± 0.15^c+^	02.32 ± 0.06^d^	2.41 ± 0.13
VI	02.59 ± 0.03	00.64 ± 0.02	02.29 ± 0.03	02.90 ± 0.30	2.28 ± 0.04

^a+,b+^ and  ^c+^ represent significant increases at p<0.05, p<0.01, and p<0.001, respectively, when compared to Group I values, while  ^d^ represents a significant decrease at p<0.05 when compared to Group I values.

I = 10 ml/kg/day of distilled water.

II = 0.3 mg/kg/day of clomiphene dissolved in distilled water.

III = 100 mg/kg/day of *Hunteria umbellata* aqueous seed extract dissolved in distilled water.

IV = 200 mg/kg/day of *Hunteria umbellata* aqueous seed extract dissolved in distilled water.

V = 400 mg/kg/day of *Hunteria umbellata* aqueous seed extract dissolved in distilled water.

VI = 20 mg/kg/day of Vitamin C dissolved in distilled water.

**Table 5:** Effect of repeated daily oral treatments with 100-400 mg/kg/day of *Hunteria umbellata* aqueous seed extract on testicular SOD, CAT, and TBARS of treated rats.

Groups	SOD (U/mg protein)	CAT (U/mg protein)	TBARS (nM/min/mg protein)
I	05.48 ± 0.11	20.77 ± 0.20	13.46 ± 0.06
II	05.37 ± 0.11	20.42 ± 0.19	13.45 ± 0.07
III	05.59 ± 0.08	20.79 ± 0.15	13.36 ± 0.08
IV	06.00 ± 0.08^c+^	21.37 ± 0.21^a+^	13.97 ± 0.06^c+^
V	06.25 ± 0.02^c+^	22.74 ± 0.03^c+^	14.49 ± 0.12^c+^
VI	06.92 ± 0.04^c+^	23.51 ± 0.16^c+^	16.89 ± 0.06^c+^

^a+^ and  ^c+^ represent significant increases at p<0.05 and p<0.001, respectively, when compared to Group I values.

I = 10 ml/kg/day of distilled water.

II = 0.3 mg/kg/day of clomiphene dissolved in distilled water.

III = 100 mg/kg/day of *Hunteria umbellata* aqueous seed extract dissolved in distilled water.

IV = 200 mg/kg/day of *Hunteria umbellata* aqueous seed extract dissolved in distilled water.

V = 400 mg/kg/day of *Hunteria umbellata* aqueous seed extract dissolved in distilled water.

VI = 20 mg/kg/day of Vitamin C dissolved in distilled water.

**Table 6:** Effect of repeated daily oral treatments with 100-400 mg/kg/day of *Hunteria umbellata* aqueous seed extract on testicular GSH, GSH-Px and GSR of treated rats.

Groups	GSH (*μ*M/g tissue)	GSH-Px (nM/mg protein)	GSR (nM/min/mg protein)
I	0.36 ± 0.02	140.00 ± 1.09	207.00 ± 2.05
II	0.35 ± 0.02	137.60 ± 1.61	203.20 ± 1.28
III	0.40 ± 0.02	139.10 ± 1.08	204.40 ± 1.80
IV	0.59 ± 0.01^c+^	143.30 ± 0.53^a+^	215.00 ± 2.22^b+^
V	0.69 ± 0.05^c+^	144.10 ± 0.17^a+^	217.80 ± 0.67^c+^
VI	0.76 ± 0.03^c+^	148.10 ± 0.66^a+^	230.60 ± 1.71^c+^

^a+,b+^ and  ^c+^ represent significant increases at p<0.05, p<0.01, and p<0.001, respectively, when compared to Group I values.

I = 10 ml/kg/day of distilled water.

II = 0.3 mg/kg/day of clomiphene dissolved in distilled water.

III = 100 mg/kg/day of *Hunteria umbellata* aqueous seed extract dissolved in distilled water.

IV = 200 mg/kg/day of *Hunteria umbellata* aqueous seed extract dissolved in distilled water.

V = 400 mg/kg/day of *Hunteria umbellata* aqueous seed extract dissolved in distilled water.

VI = 20 mg/kg/day of Vitamin C dissolved in distilled water.

## Data Availability

The data used to support the findings of this study are available from the corresponding author upon request.
